# Evaluation of peripheral auditory pathways and brainstem in obstructive sleep apnea^☆^

**DOI:** 10.1016/j.bjorl.2016.10.014

**Published:** 2016-11-25

**Authors:** Erika Matsumura, Carla Gentile Matas, Fernanda Cristina Leite Magliaro, Raquel Meirelles Pedreño, Geraldo Lorenzi-Filho, Seisse Gabriela Gandolfi Sanches, Renata Mota Mamede Carvallo

**Affiliations:** a Universidade de São Paulo (USP), Faculdade de Medicina, Departamento de Fonoaudiologia, Fisioterapia e Terapia Ocupacional, São Paulo, SP, Brazil; b Universidade de São Paulo (USP), Faculdade de Medicina, Laboratório do Sono, Divisão de Pneumologia, Instituto do Coração (InCor), São Paulo, SP, Brazil

**Keywords:** Obstructive sleep apnea, Hypoxia, Auditory brainstem response, Hearing test, Apneia obstrutiva do sono, Hipóxia, Potenciais evocados auditivos do tronco encefálico, Audição

## Abstract

**Introduction:**

Obstructive sleep apnea causes changes in normal sleep architecture, fragmenting it chronically with intermittent hypoxia, leading to serious health consequences in the long term. It is believed that the occurrence of respiratory events during sleep, such as apnea and hypopnea, can impair the transmission of nerve impulses along the auditory pathway that are highly dependent on the supply of oxygen. However, this association is not well established in the literature.

**Objective:**

To compare the evaluation of peripheral auditory pathway and brainstem among individuals with and without obstructive sleep apnea.

**Methods:**

The sample consisted of 38 adult males, mean age of 35.8 (±7.2), divided into four groups matched for age and Body Mass Index. The groups were classified based on polysomnography in: control (*n* = 10), mild obstructive sleep apnea (*n* = 11) moderate obstructive sleep apnea (*n* = 8) and severe obstructive sleep apnea (*n* = 9). All study subjects denied a history of risk for hearing loss and underwent audiometry, tympanometry, acoustic reflex and Brainstem Auditory Evoked Response. Statistical analyses were performed using three-factor ANOVA, 2-factor ANOVA, chi-square test, and Fisher's exact test. The significance level for all tests was 5%.

**Results:**

There was no difference between the groups for hearing thresholds, tympanometry and evaluated Brainstem Auditory Evoked Response parameters. An association was observed between the presence of obstructive sleep apnea and changes in absolute latency of wave V (*p* = 0.03). There was an association between moderate obstructive sleep apnea and change of the latency of wave V (*p* = 0.01).

**Conclusion:**

The presence of obstructive sleep apnea is associated with changes in nerve conduction of acoustic stimuli in the auditory pathway in the brainstem. The increase in obstructive sleep apnea severity does not promote worsening of responses assessed by audiometry, tympanometry and Brainstem Auditory Evoked Response.

## Resumo

**Introdução:**

A Apneia Obstrutiva do Sono provoca modificações na arquitetura normal do sono, fragmentando-o de forma crônica com hipóxias intermitentes levando, a longo prazo, a sérias consequências na saúde. Acredita-se que a ocorrência de eventos respiratórios durante o sono como apneia e hipopneia pode prejudicar a transmissão de impulsos nervosos ao longo da via auditiva que são altamente dependentes do fornecimento do oxigênio. Contudo, essa associação não se encontra bem estabelecida na literatura.

**Objetivo:**

Comparar os achados da avaliação da via auditiva periférica e no tronco encefálico entre indivíduos portadores e não portadores de apneia obstrutiva do sono.

**Método:**

A casuística foi composta por 38 adultos do sexo masculino, média de idade de 35,8 (±7,2); divididos em quatro grupos experimentais pareados por idade e índice da massa corpórea. Os grupos foram classificados com base na polissonografia em: controle (n = 10), apneia obstrutiva do sono leve (n = 11), apneia obstrutiva do sono moderada (n = 8) e apneia obstrutiva do sono grave (n = 9). Todos os sujeitos do estudo negaram história pregressa de risco para perda auditiva e foram submetidos à audiometria, timpanometria, pesquisa dos reflexos acústicos e Potenciais Evocados Auditivos de Tronco Encefálico. As análises estatísticas foram realizadas por meio de ANOVA 3-fatores, ANOVA 2-fatores, teste de Qui-quadrado e teste exato de Fisher. O nível de significância adotado para todos os testes foi de 5%.

**Resultados:**

Não houve diferença entre os grupos para os limiares auditivos, timpanometria e parâmetros avaliados do Peate. Observou-se associação entre a presença da apneia obstrutiva do sono e alteração da latência absoluta da onda V (*p* = 0,03). Observou-se associação entre apneia obstrutiva do sono de grau moderado e alteração da latência da onda V (*p* = 0,01).

**Conclusão:**

A presença de apneia obstrutiva do sono está associada à presença de alteração na condução nervosa do estímulo acústico na via auditiva em tronco encefálico. O aumento do grau de severidade da apneia obstrutiva do sono não promove piora das respostas avaliadas pela audiometria, timpanometria e Potenciais Evocados Auditivos de Tronco Encefálico.

## Introduction

Obstructive sleep apnea (OSA) is recognized as one of the major causes of morbidity and mortality, and is associated with a wide range of cardiovascular, metabolic, neurological, physiological changes, as well as patient cognitive impairments, and has been considered as one of the major problems of public health.^1^, ^2^, ^3^, ^4^, ^5^, ^6^ A recent study of adults performed in the metropolitan area of the city of São Paulo showed that the prevalence of Obstructive Sleep Apnea Syndrome (OSAS) is high and increasing, currently around 33%.^5^

The possibility of OSA interfering with the process of generation and transmission of nerve impulses in the auditory system is reported by previous studies, but this association is not well established, and there is doubt as to the actual effect of OSA on hearing.^7^, ^8^ In addition, individuals with OSA could have serious changes in the mechanisms described above, due to the hyperviscosity of blood plasma^9^ and the hypoxic cycles present in OSA.^10^

This study aimed to compare the findings of the evaluation of the peripheral auditory pathways and of the brainstem among individuals with and without OSA.

## Methods

This study was approved by the Institutional Ethics Committee under protocol number 1.437.604, and all volunteers agreed to participate in the study by signing the Informed Consent before undergoing evaluation.

The sample consisted of 38 adult males divided into four groups: control (*n* = 10), mild OSA (*n* = 11), moderate OSA (*n* = 8), and severe OSA (*n* = 9). All of them were matched for age and Body Mass Index (BMI). All subjects underwent polysomnography. This was followed by the following classification of the American Academy of Sleep Medicine for the severity of OSA considering the Apnea and Hypopnea index (AHI) per hour of sleep: mild OSA (AHI between 5 and 15 events per hour of sleep); moderate OSA (AHI between 15 and 30 events per hour of sleep); and severe OSA (AHI ≥30 events per hour of sleep).^11^

The exclusion criteria adopted in the study were: Body Mass Index (BMI) greater than or equal to 40 kg/m^2^, treatment for OSA with continuous positive airway pressure machine (CPAP) or intraoral devices, heart failure, diabetes, hypertension, thyroid changes, dyslipidemia, stroke and a history of risk for hearing loss, including exposure to occupational noise.

**Figure 1:**
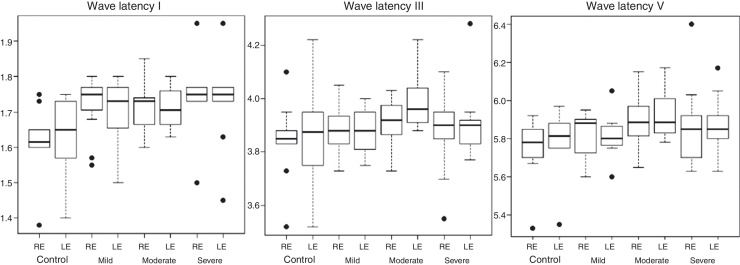
Absolute latencies of waves I, III and V (in milliseconds) according to the side of the ear and group.

**Figure 2:**
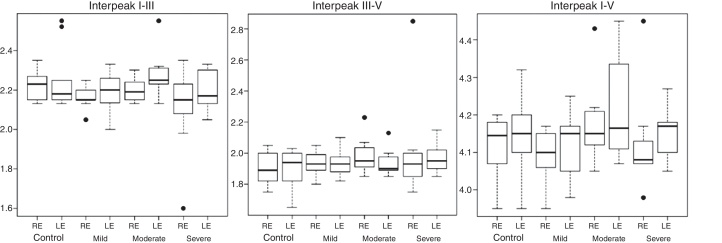
Interpeaks I–III, III–V and I–V (in milliseconds) according to the side of the ear and group.

Conventional threshold tonal audiometry was investigated in the frequencies of 250–8000 Hz through the GSI-61 equipment (Grason Stadler, United States) and TDH 50P supra aural headphones (Telephonics, United States). For the investigation of tympanometry, the tympanometry curve of admittance was recorded with a 226-Hz probe tone, and ipsilateral and contralateral acoustic reflex investigation at frequencies of 500, 1000, 2000 and 4000 Hz. Tympanometry and acoustic reflexes evaluations were performed through the Titan equipment (Interacoustics, Denmark). The evaluation of Brainstem Auditory Evoked Response (BAER) was performed using Smart EP (Intelligent Hearing Systems, United States). The acoustic stimulus used to record BAER was the click with rarefied polarity (with 100-Hz high pass filter and 3000-Hz low pass) monoaurally presented at 80 dBnHL with time of stimulus of 0.1 ms, and presentation frequency of 19.1 stimuli per second. In the total, 2048 stimuli were presented, with the number of artifacts being always less than 10% of the stimuli presented. The stimulus with condensed polarity was employed in situations where the amplitudes of the responses showed to be reduced, allowing the selection of the best route of waves for analysis. Two tracings were recorded to ensure wave reproducibility. The values of absolute latencies of waves I, III and V and interpeaks I–III, III–V and I–V in milliseconds (ms) were identified and quantitatively analyzed (Figure 1, Figure 2).

BAER results were classified as normal or abnormal on each side of the ear. The normal range of values of absolute latencies of waves I, III and V, and interpeak I–V proposed by the Smart EP equipment manual – Intelligent Hearing Systems^12^ (Table 1) was adopted.

**Table 1:** Pattern of normality of latency and interpeak values of BAER for adults, proposed by the manual of Smart EP – IHS.

	Wave I	Wave III	Wave V	Interpeak I–V
Average (ms)	1.59	3.64	5.57	3.98
Standard deviation (ms)	0.24	0.17	0.16	0.25

Two values of standard deviation are considered for the classification of normal latency.

Kruskal–Wallis nonparametric test was used to evaluate the homogeneity among the groups of baseline variables such as age, Body Mass Index (BMI) and Apnea and Hypopnea index (AHI). Models of analysis of variance (ANOVA) of 3 factors^13^ for audiometry and ipsilateral and contralateral acoustic reflexes were used. In the models, the factors disease group (Group), ear side (Ear) and Frequency were considered, with the latter two factors being considered with repeated measures. Due to the large number of descriptive levels associated with the Frequency factor, the correction proposed by Greenhouse–Geisser was applied.^14^ For tympanometry parameters, Tympanometric Peak Pressure (TPP), and admittance compensated at the level of the tympanic membrane (Y_TM_) and BAER (latencies of waves I, III and V and interpeaks I–III, III–V and I–V), models of 2-factors ANOVA were used. For multiple comparisons the method proposed by Holm was used.^15^

To verify the association between the degree of OSA severity and the number of ears with responses out of normal standard for latencies I, III and V, and interpeak I–V, a nonparametric chi-square test was performed. Through Fisher's exact nonparametric test, multiple comparisons were performed of the number of ears with responses out of normal standard for latencies I, III and V, and interpeak I–V of BAER of each disease group (mild, moderate and severe), confronting them with the control group.

All statistical analyses were performed using the R software version 3.2.2 (R Foundation, United States) and Minitab 17 (Minitab Inc., United States). The significance level for all tests was 5% (*α* = 0.05).

## Results

### Sample characterization

The study included a total of 38 subjects, mean age of 35.3 (±7.1) years and mean BMI of 28.8 (±3.8) kg/m^2^. As expected, no significant differences were observed among disease groups according to age (*p* = 0.08) and BMI (*p* = 0.15). In addition, significant difference was found for AHI (*p* < 0.001), indicating that all groups in this study were statistically different.

### Audiometry, tympanometry and acoustic reflexes

The average hearing thresholds did not differ among the groups (0.22). There was difference only between the frequencies tested in audiometry (*p* = 0.002), with higher frequencies (4, 6 and 8 kHz) presenting higher thresholds than at 3 kHz. For tympanometric parameters of TPP and Y_TM_, it was not possible to detect any significant difference. Furthermore, the values for the ipsilateral acoustic reflexes do not differ among the groups, nor in any other factors analyzed. The results for the contralateral acoustic reflex, on their turn, showed difference only for the Frequency factor (*p* < 0.001). That is, the average of the frequencies of 500, 1000 and 4000 Hz were higher than the average frequency of 2000 Hz.

### Brainstem auditory evoked potential

There was no statistical difference for any of the effects of interaction between the factors Group and Ear in the BAER parameters evaluated. There was no significant difference for the main effects of the factors Group and Ear. Thus, for BAER variables, there were no significant differences among the averages of the groups and among the means of the ears. There was no significant difference between the groups (*p* = 0.8517). Figure 1, Figure 2 show the boxplot graphics for the absolute latencies of waves I, III and V, and interpeaks I–III, III–V and I–V, according to the side of the ear and group.

Table 2 shows the percentage of changes found in each group in relation to the absolute latencies of waves I, III, V and interpeak I–V, taking the normal range^12^ provided by the equipment used in the study into account. The chi-square test showed an association between the presence of OSA and a change in absolute latency of wave V (*p* = 0.03). Considering the degree of severity of OSA, Fisher's exact test found the association between the presence of moderate OSA and the presence of change in absolute latency of wave V.

**Table 2:** Changes of latencies of waves I, III, V and interpeak I–V for each ear of each group considering the normal values provided by Smart EP – Intelligent Hearing Systems.

BAER parameters	Percentage of change compared to the number of ears evaluated				*p*-value^a^
	Control *n* (%)	Mild OSA *n* (%)	Moderate OSA *n* (%)	Severe OSA *n* (%)	
Absolute latency of wave I	0	0	0	0	–
Absolute latency of wave III	3 (15)	4 (18.2)	6 (37.5)	2 (11.1)	0.23
Absolute latency of wave V	2 (10)	4 (18.2)	8 (50)	6 (33.3)	**0.03**
Interpeak I–V	1 (5)	1 (4.55)	5 (31.25)	3 (16.7)	0.07
*p*-value^b^	–	0.76	**0.02**	0.17	–

a Chi-square test.

b Comparison of each group with OSA (mild, moderate and severe) with the Control group only for absolute latency of wave V through Fisher exact test. The significance level for all tests was 5% (*α* = 0.05).

## Discussion

Regarding baseline variables, it is observed that the four study groups are properly matched for age and BMI. The age range of the sample of this study was wide. However, many studies involving the hearing of individuals with OSA have also included samples in a similar way^8^, ^9^, ^16^ and even greater than that found in this study^17^, ^18^, ^19^ (Table 3).

**Table 3:** Sample age range in studies relating OSA and hearing compared to this study.

Studies	Age range (mean/standard deviation)	Total *n* (male *n*)	OSA *n* (control N)
Mosko et al., 1981	24–40 (31 ± 2)	20 (12)	6 (14)
Hoffstein et al., 1999	13–74 (47 ± 12)^a^	219 (156)	–
Bernath et al., 2009	30–55 (48)^b^	610 (610)	610
Gallina et al., 2010	24–56 (43)	75 (51)	45 (30)
Casale et al., 2012	25–45 (31)^b^	39 (31)	18 (21)
Sivri et al., 2013	27–66 (47 ± 6.23)^b^	138 (96)	78 (60)
Ballacchino et al., 2015	14–85 (57 ± 13.15)	120 (77)	c
Thom et al., 2015	37–74 (57)	10 (6)	10
Present study	22–54 (35.3 ± 7.1)	38	28 (10)

a Mean age and standard deviation of males with OSA.

b Mean age of study group with OSA.

c Subjects with risk of OSA. –, not specified.

Regarding audiometry, no statistical difference in auditory thresholds caused by the presence of OSA was observed. Specifically, a subject of the group with severe OSA had mild hearing loss^20^ in the left ear, whose average of hearing thresholds of 500, 1000 and 2000 Hz was 26.66 dBHL. Moreover, few individuals had low auditory threshold in isolated frequencies: 1 individual from the control group and 3 individuals in the group with severe degree of AOS. A similar result was observed in another study,^16^ in which the presence of low auditory threshold was found, specifically in control subjects without OSA, and also without reporting a history of hearing loss. Unlike the present study, another study^18^ found that individuals at high risk for the presence of OSA had lowered tone thresholds when compared to control. However, for the above study, the risk for OSA was assessed through a standardized questionnaire, with no polysomnography diagnosis. Also, the fact that a very wide age range was selected (14–85 years) with the presence of tinnitus may have contributed to the difference in the hearing threshold, since the two factors are strongly related to the manifestation of hearing loss.^21^, ^22^

Regarding tympanometry, the results were considered normal for adults,^23^, ^24^ probably due to the exclusion criteria adopted in this study. These data did not agree with the findings of the literature where tympanometric curves of types B and C were observed in subjects with moderate and severe degree of OSA.^25^ This can be explained by the fact that the study cited have included individuals who had undergone septoplasty three months before the hearing evaluation.^25^

BAER examination provides objective information that can be considered as parameters for diagnostic purposes, as for the structural and functional integrity of the auditory pathways to the brainstem, being widely used clinically, both because it has well-defined generators, and due to the characteristic of reproducibility.^26^ According to a study, adults with OSA have high viscosity of blood plasma, with this hyperviscosity being positively correlated with OSA severity.^27^ The hyperviscosity and its consequent change of microcirculation were considered the main causes for changes in BAER in adults with OSA.^9^ These studies suggest that individuals who have OSA disease, when not treated with a continuous positive airway pressure device (CPAP), for example, have blood hyperviscosity. In addition, intermittent hypoxia present in adults with a severe degree of OSA may represent a risk factor for impairment of the auditory pathway, and the severity and/or duration of OSA may contribute to the decrease of neuronal and vascular function of the auditory pathway.^8^ Therefore, changes were expected in the auditory pathway in adults with OSA, according to the disease severity.

In this study, the analysis of qualitative data showed an association between the presence of OSA and a change in absolute latency of wave V of BAER (Table 2). Moreover, considering the multiple comparisons of groups with disease compared to the control, the group with moderate OSA showed an association with change in latency of wave V. Although not significant, it is important to consider that the group with severe OSA had an increased percentage (33%) of change in wave V latency compared to the control (10%). Likewise, the groups with higher severity of OSA, moderate and severe, had greater occurrence of change in interpeak I-V compared to the control.

The study shows that individuals with severe hypoxemia or hypercapnia have high prevalence of abnormalities in BAER.^28^ Similar results have been reported, showing increased interpeak I–V in 36.5% of subjects with OSA.^16^ Casale et al. found changes in interpeaks III–V and I–V in the group with severe degree of OSA, showing the presence of change in retrocochlear auditory pathways.^8^ The same authors also identified increased values of latencies I, III, V and interpeaks III–V and I–V in severe degree of OSA compared to control.^8^ In another study, subjects with OSA and hyperviscosity showed impaired auditory pathway in the brainstem from the caudal portion to the superior olivary complex.^9^ These changes may be present as a result of reduced blood flow in the brainstem region that has been observed in patients with apnea and that, therefore, could be exacerbating the harmful effects of apnea in the auditory pathway of the brainstem.^29^ Additionally, the resulting modified microcirculation of blood hyperviscosity appears as the main cause of changes in BAER in individuals with OSA.^9^

The reversibility of the change in BAER (latency wave III normalized) has been reported in adults with OSA who were treated and had blood viscosity normalized. The same authors speculate that patients with OSA without hyperviscosity, with results within normal standards in BAER, have effective compensatory mechanisms that balance hypoxemia, hypercapnia, acidosis and altered microcirculation caused during apnea.

There are studies^30^, ^31^ that showed discordant results compared to this study, since no change was found in the brainstem in individuals with OSA syndrome. However, the same authors^30^, ^31^ conducted case studies with a considerably small sample with eleven and six subjects with OSA, respectively. This study did not observe any difference among the values obtained in BAER parameters according to the severity of OSA. However, the power of the statistical test for the quantitative analyses of BAER in this sample of 38 adults was 80% only for large effects, which was considered as a limiting factor. In addition, the presence of the control group with changes in absolute latencies of waves I, III, V, and interpeaks I–III, III–V and I–V of BAER, may have hindered the more precise identification that would confirm the hypothesis that OSA influences the auditory responses in different generating sites of the auditory pathway, according to the degree of severity of the disease.

As the available literature that involves the study of BAER in OSA is scarce and shows an even inconsistent scenario, precluding the establishment of the real influence of OSA in the structural and functional integrity of the auditory pathway, new studies to clarify the association of OSA with the damage of the auditory pathway in the brainstem are necessary.

## Conclusion

The presence of OSA is associated with the presence of changes in nerve conduction of acoustic stimuli in the auditory pathway in the brainstem. The increase in OSA severity does not promote the worsening of responses assessed by audiometry, tympanometry and BAER.

## Funding

This study received financial support from the São Paulo Research Foundation (FAPESP 2013/10281-7).

## Conflicts of interest

The authors declare no conflicts of interest.
